# Views from Within a Narrative: Evaluating Long-Term Human–Robot Interaction in a Naturalistic Environment Using Open-Ended Scenarios

**DOI:** 10.1007/s12559-014-9284-x

**Published:** 2014-11-06

**Authors:** Dag Sverre Syrdal, Kerstin Dautenhahn, Kheng Lee Koay, Wan Ching Ho

**Affiliations:** Adaptive Systems Research Group, School of Computer Science, University of Hertfordshire, Hatfield, UK

**Keywords:** Human–robot interaction, Prototyping, Assistive robotics

## Abstract

This article describes the prototyping of human–robot interactions in the University of Hertfordshire (UH) Robot House. Twelve participants took part in a long-term study in which they interacted with robots in the UH Robot House once a week for a period of 10 weeks. A prototyping method using the narrative framing technique allowed participants to engage with the robots in episodic interactions that were framed using narrative to convey the impression of a continuous long-term interaction. The goal was to examine how participants responded to the scenarios and the robots as well as specific robot behaviours, such as agent migration and expressive behaviours. Evaluation of the robots and the scenarios were elicited using several measures, including the standardised System Usability Scale, an ad hoc Scenario Acceptance Scale, as well as single-item Likert scales, open-ended questionnaire items and a debriefing interview. Results suggest that participants felt that the use of this prototyping technique allowed them insight into the use of the robot, and that they accepted the use of the robot within the scenario.

## Introduction

This article describes the prototyping of long-term human–robot interactions with companion robots in a domestic environment through the use of episodic, narratively framed interactions.

The work described in this article was performed as part of the LIREC (LIving with Robots and intEractive Companions) [1] and ACCOMPANY (Acceptable robotiCs COMPanions for AgeiNg Years) [2] projects. Both of these projects focus on the use of complex multi-role autonomous companion robots in human-centred environments.

Prototyping such robots in domestic environments can be very challenging, especially if it is to be performed with research platforms that are still under development. While research using these platforms allows for prototyping that is at a stage early enough for the results to have meaningful impact on the technological development of a project [3], or, in the case of basic research, to the direction of a research field, such platforms are often inherently unstable. This instability impedes the platform’s ability to function beyond a short amount of time without continuous oversight and maintenance by trained technical personnel. In the case of mobile robots, or robots with object manipulation capabilities, this may pose a serious safety concern. These issues may render their deployment with members of the general public for human–robot interaction testing purposes problematic, and often impossible. Because of this, the majority of the research on domestic robots in the home environments of their users has focused either on products that are market-ready, or approaching this status [4–8], or on robots that function as fully embodied conversational agents [9] (similar to screen-based agents such as in [10]).

Dautenhahn [11] acknowledges this difficulty and argues that these particular problems for the field of human–robot interaction (HRI) as a whole make pragmatism and creativity in terms of methodology paramount, and much of our work is centred around how one can meaningfully prototype interactions with such robots in a safe manner that still allow potential users insight into the experience of using the system in settings for which it is intended.

In this paper, we present a case study on prototyping such meaningful interactions as part of long-term study in a domestic environment. Empirical results as well as methodological challenges are discussed. In order to do so, we will outline the background for our work in the “Prototype Fidelity in Human–Robot Interaction” section, which introduces the issue of prototype fidelity when considering human–robot interaction prototyping. The “Narrative Framing for Contextual Fidelity” section outlines our general approach to responding to this problem when prototyping interactions with robots in the University of Hertfordshire Robot House. Finally, the “From Usage to Evaluation Scenarios” section provides an example of how narrative framing techniques (a concept adapted from [12]) are used to perform high-fidelity interaction prototyping with naïve users.

### Prototype Fidelity in Human–Robot Interaction

When considering how different prototyping methods vary from each other, one pertinent dimension is that of *fidelity,* defined by Hall [13] as “faithfulness in reproducing the characteristics of the finished product” (ibid, p. 491). When comparing the fidelity of robotic prototypes to that of software prototypes, there are some clear differences. One argument that has been put forward in HRI for human-centred environments is that the novelty of the systems used requires a high degree of fidelity when prototyping [14]. This view is echoed to some extent by Bartneck [15], who also puts forward the three-dimensional, embodied nature of robots and the spatial and tactile interaction affordances. Bartneck [15] also highlights that the complexity of robotic systems makes the issue of fidelity less clear cut than that of software systems. We consider the fidelity of prototyping for the LIREC and ACCOMPANY projects to have two main dimensions:

- Fidelity of platform
- Fidelity of setting

#### Fidelity of Platform

The fidelity of the robot may vary widely, and we can roughly consider it along two dimensions. One is the physical richness of the prototype. On the low end, we may here consider some studies that have been performed on robots and devices that are only realised in written stories [16, 17] with videos of robots being considered a step up in terms of fidelity [18, 19]. Theatre plays in which actors either pretend to be [20] or interact with actual robots in a space shared with the audience [21, 22] could here be considered the highest level of fidelity apart from actual interactions with physically embodied robots.

However, one should also consider the fidelity of such systems in terms of the realism of their behaviour. This comprises not only the degree that their behaviour reflects the projected behaviour of the completed technology, but also the degree in which the system is capable of producing these behaviours without being controlled by its developers. A common technique in HRI is the so-called Wizard of Oz (WoZ) methodology [23], in which the robot portrays seemingly autonomous behaviours, allowing researchers to bypass issues that make it difficult to run the system autonomously. It has been argued, however, that reliance on this methodology comes with serious problems, in particular that it poses a problem due to the possibility of it creating unrealistic interactions and findings that are not grounded in a realistic interaction between users and systems, which in turn threaten the validity of such studies [24].

#### Fidelity of Setting

Fidelity of setting can also be understood as ecological validity. By this, we mean to what extent the context in which an interaction takes place is applicable to the context in which a robot will actually be used in the future. As for the fidelity of the system, this is not a unidimensional construct. In our current work, we see the fidelity of setting as having two dimensions, physical and contextual. Both may impact the nature of the participant’s experience of the system and their subsequent evaluation.

For instance, Walters et al. [25] describe a study on participants’ proxemic expectations of a robot and the relationship between these and their subsequent evaluation of the robot, in a constrained experiment in the University of Hertfordshire Robot House (see below). The setting and environment could be considered high in terms of physical fidelity in the sense that the participants were interacting with an actual robot and were capable of responding to the physicality of the robot, in a physical environment that was similar to that in which such interactions are envisaged to take place.

Lohse et al. [18] describe a study in which participants watched video interactions had by a user with their own robot in their own home and were then invited to share their thoughts and opinions about what they had seen. In this study, despite the lack of physical interactivity, users were exposed to a rich and meaningful scenario in which they could see the impact of the robot on the user’s everyday experience, thus allowing the participants to understand the role of the robot in its intended setting. However, this was a setting not shared by the participants who only experienced it vicariously.

While we acknowledge that both of these studies provided the researchers with valuable insights, they also illustrate the importance of tying both the level of fidelity and the type of prototype used, to the research objectives of the study [26].

### Narrative Framing for Contextual Fidelity

Our work in the LIREC and ACCOMPANY projects focuses on the holistic experience of our participants when interacting with robots in real-life domestic settings. Because of this, we want to present our participants with physical prototypes that behave realistically in a setting which is clearly applicable to the use scenarios of a proposed robotic companion. We have previously proposed the UH Robot House as an ecologically valid test bed for HRI studies, as it is a residential house that has subsequently been adapted for such studies [25].

The UH Robot House is furnished as a normal British house, but is also used for technical development in the domains of smart home technology and robot-assisted living. This means that it is equipped with a low-cost, resource-efficient sensor network which can be used to detect and keep track of user activities and other events in the environment [27]. The autonomous robots used for HRI studies in the house are an integral part of this smart home. The robot house has been used with a range of robots such as the UH Sunflower Robot [28], Mobile Robots’ PeopleBots [25] and the Fraunhofer IPA Care-O-bot^®^ 3 [29]. This allows for a setting with high-fidelity prototypes both in terms of physicality as well as in behaviour realism.

This setting has allowed us a solid starting point to address the issue of *contextual* setting fidelity. While there have been instances of artists using the UH Robot House continuously for 5 days [30], the robots and the smart home technology are not stable enough to allow for 24/7 residency by members of the general public, even though this would be desirable for extensive user testing of the system. Because of this, we decided on applying a *narrative framing technique* for prototyping using episodic interactions in which we use *narrative* to frame each individual interaction [12]. This would allow us to draw on the usage scenario as the basis for the narrative, using the robots and the house itself as props for the emergent interactions.

It is important for this process that the UH Robot House is a working house, with kitchen appliances that can be used to cook, a TV that can be used to relax, a doorbell that rings when visitors arrive and so on. This will allow the users to actually perform activities that are congruent with the interaction scenarios envisaged by the researchers.

#### Previous Work in the Robot House

We have previously conducted studies using similar methodologies, where we performed a series of episodic interactions within the UH Robot House [31] and used similar narrative framing techniques for setting the scene for the different episodes. This allowed us to examine participant responses to a variety of robot behaviours, as well as allowing the participant the chance to consider wider implications of domestic robots [32]. Note, in this previous work, a smaller Robot House was used (a ground-floor flat), without a sensor network and with the robots controlled primarily via WoZ.

These previous studies were useful for examining the role of habituation in responses to some robot behaviours, as well as providing experience in running such studies away from the confines of the laboratory, but they also suffered from some limitations. The most serious of these was the fracturing of the role of the participant and the robot. While in some of the episodes the participant was asked to take on the role of a robot owner in their own house, in others they were asked to take on the role of a guest [32], teacher [33] or even co-designer of robot behaviours [34]. One side effect of this fracturing was that the participants could never be sure, in a sense, “what” robot house they were visiting. Was it a house in which they were the active owner, going about their daily business, or was it a house where they were visiting a robot owner, or indeed not a private house at all, but rather a workshop where robot designers elicited their help? Similarly, since the robots were partially remotely controlled by a present researcher, the role of the robot and the researchers were likewise fractured.

This uncertainty regarding roles might have been an impediment to the participants’ ability to evaluate the robot and its possible roles outside of the experimental setting, within their everyday lives and beyond the scope of the individual interaction episode. In the present study, we intended to overcome these limitations of our earlier work.

#### Requirements of the Prototyping

Based on this previous work, we arrived at the following requirements for our current study:

- Coherent narrative—The participants need to feel that they are interacting with the same system in the same setting in the open-ended scenarios.
  - Realised through:
    - Using the same interface throughout the study
    - The environment is kept stable
    - The participant is always the “owner” of the house
    - It is made clear to the participant when they are “inside” the narrative
- Agency—The participants need to have a clear idea about what they “want” to achieve in a session as well as how this can be achieved.
  - Addressed by making sure participants:
    - Understand the interface of the robot
    - Understand the workings of the house
    - Know locations of items used in the scenarios
    - Understand how to use the appliances
- Realistic system—The behaviour of the system should reflect its true capabilities.
  - Reflected through:
    - Scenarios being based on the system’s actual capabilities (autonomously operating smart home)
    - Human technicians monitor the functioning of the system and only intervene in case of faults or bugs appearing.

**Fig. 1:**
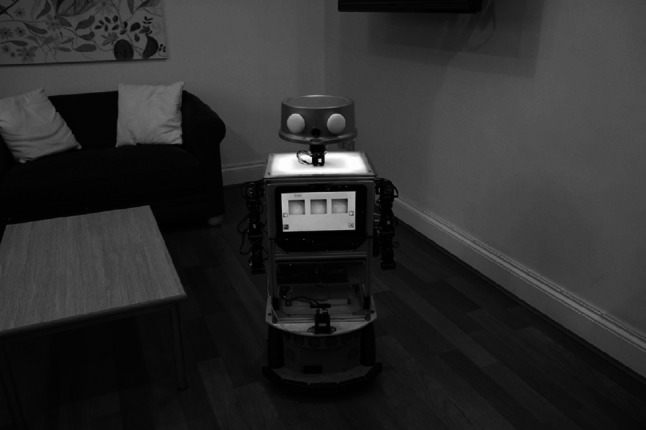
UH Sunflower robot in the University of Hertfordshire Robot House

Using these requirements to guide the development of our study, we could then proceed to investigate the role of the robot (Fig. 1) within our project scenarios, which will be considered next.

### From Usage to Evaluation Scenarios

Much of the development work within the UH Robot House is based around the lives of two constructed *personas*, i.e. highly realised fictional users (a method for design often used in HCI [35]). The specific personas used to guide development in the Robot House are a couple (David and Judy) in their mid-to-late 60s. The personas were fleshed out and realised by considering their work interests, hobbies and specific health issues that would allow us to examine the role of technology within their lives. Below is a brief introduction to the personas and the scenarios derived from them:

David is recently retired from an office-based job, in which he used computers on a daily basis. In his retirement, he is planning to focus on his hobbies. Some of these hobbies are sedentary and require little assistance, like reading and watching documentaries. He also enjoys building military models which requires him to move quite a lot of objects from storage areas to work surfaces. He also needs to take medications regularly to manage a heart condition. For some reason, he often forgets to take this medication and Judy (his wife) needs to remind him of this on a daily basis. Due to arthritis, he also has some mobility issues.

For Judy, their house is also her primary work place. She works as a consultant, which means that unless she is visiting clients, she spends most of her working hours in the home office. David’s recent retirement has led to her getting distracted more easily due to his presence in the house, and there is some tension between them because of this. Because of this, Judy now has adopted a separation of work and leisure, and keeps to her home-office during working hours, only interacting with David during mealtimes and in the evenings and weekends. Like David, she is used to computing technology, relying on it to work effectively from her home office. Unlike David, however, she is more used to solving problems related to computing technologies by herself. She also uses social media and voice communication applications to keep in touch with their children and grandchildren.

**Fig. 2:**
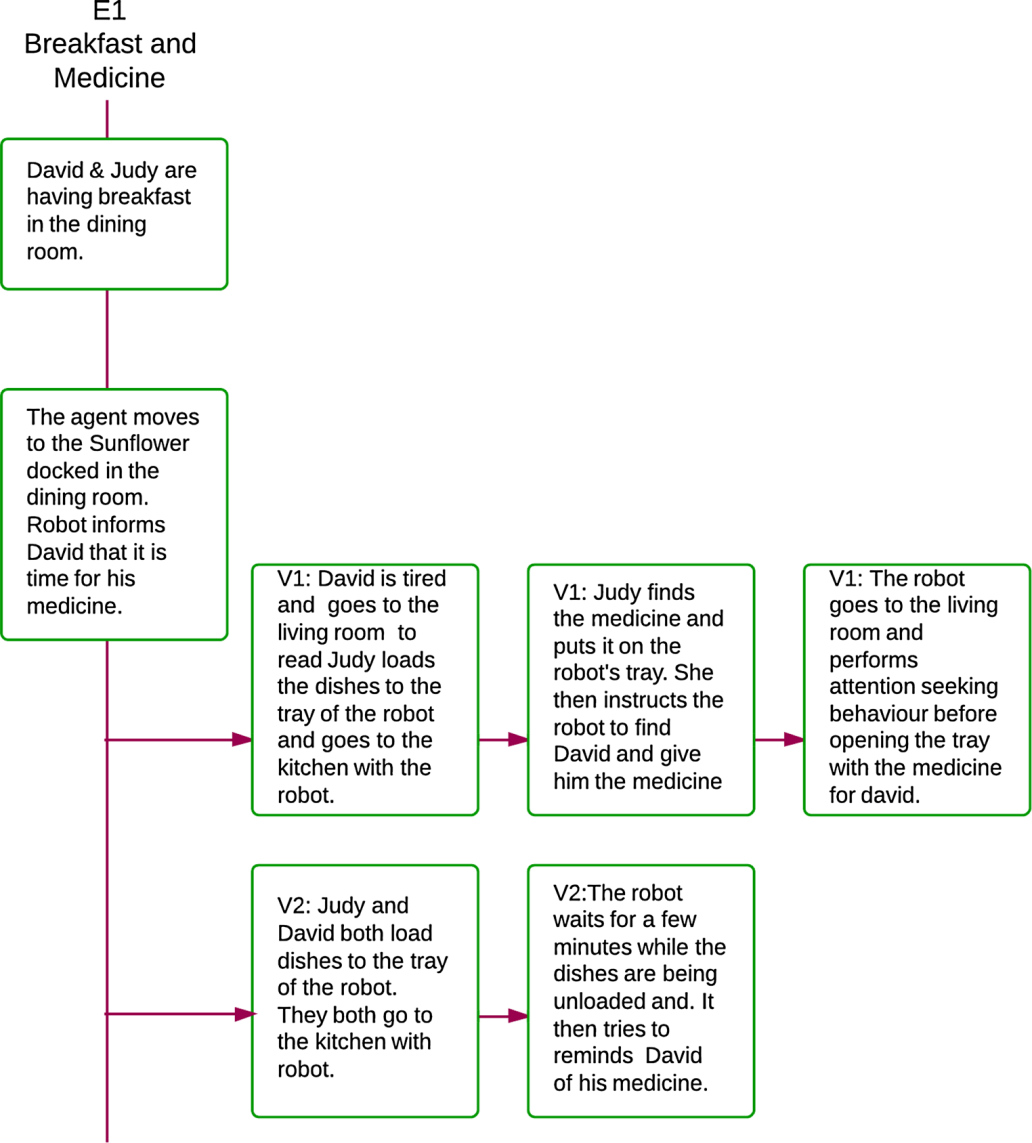
Sample episode from the usage scenario-Breakfast and Medicine

**Fig. 3:**
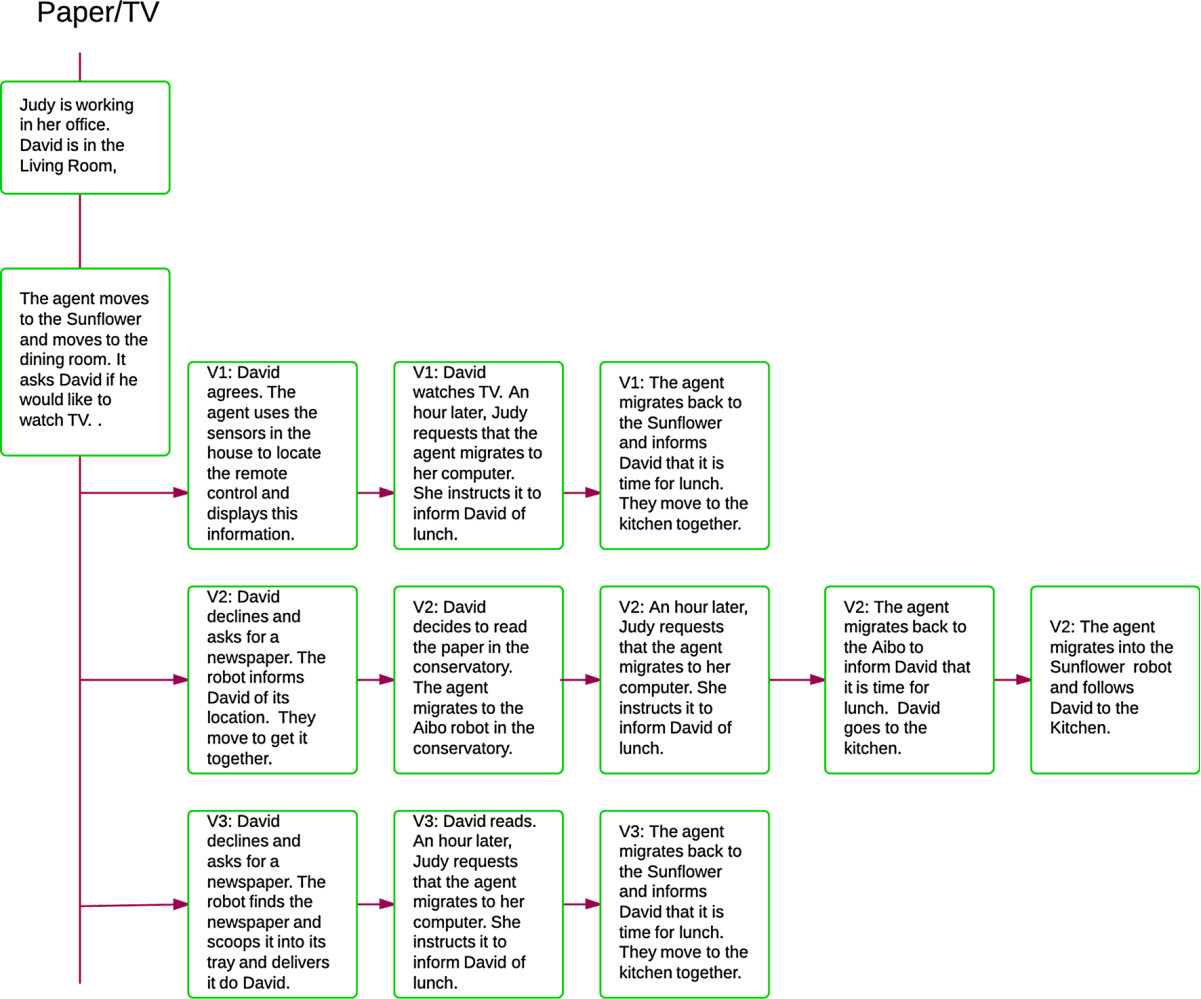
Sample episode from usage scenario-Papter/TV

Based on these personas, a “typical” day comprising of episodic usage scenarios where the couple used the robot in their normal everyday activities was created (see Figs. 2, 3 for a high-level conceptual description and a more technical description in Table 1). These episodes were then used as the basis for creating two *evaluation scenarios* where we could examine the possible roles that the robot could play in these different episodes. These were an attempt to convey the impact of the robot within a wider context. They differed from the usage scenarios in that they were intended for a single user, and would be meaningful to an experimental participant within the context of a one-hour interaction.

**Table 1:** Sample usage episodes

Scenario name	Hobby—building airfix models
Origin	User initiated
Companion embodiment	Sunflower
Chronological overview	David uses touch screen to instruct companion to follow him to the model storage area. Companion follows David to storage area. David loads models from storage area onto the robot and instructs robot to move to the dining area workspace Companion moves to the workspace David unloads models and starts working Companion waits for 1 h, then attracts David’s attention and suggests a break
Competencies	Follow user Navigation Accessing schedule for breaks Attention seeking
Scenario name	Time for lunch
Origin	Scheduled event
Companion embodiment	Embodied conversational agent (ECA), Sunflower, AIBO
Chronological overview	Companion appears on Judy’s screen as an ECA, and informs her that she has scheduled lunch for this time Companion migrates from ECA to Sunflower embodiment and follows Judy to the kitchen Judy prepares food and asks the companion to find out what David’s preferences are for this meal Companion migrates from Sunflower to AIBO to ask David about his preferences and migrates back to Sunflower to give this information to Judy Judy loads Sunflower with the plates and food from the kitchen and moves to the dining area
Competencies	Accessing schedule Migration between different embodiments Navigation Communication Attention-seeking
Scenario name	Package delivered
Origin	Sensor event
Companion embodiment	Sunflower
Chronological overview	Delivery person rings the doorbell Companion is alerted via the robot house sensors Companion migrates to Sunflower robot and searches for David Companion attracts David’s attention and informs him that there is someone at the door David and companion go to the door together
Competencies	Detecting sensor events Person finding Attention seeking Navigation

As such, they were grounded in an imagined daily life. This notion was supported by allowing the participant to inform the robot about their preferences in terms of drinks, snacks and leisure activities, and TV programmes that they preferred in their own daily life prior to the first interaction with the robot. Subsequent interactions with the robot would then draw on these in order to convey a sense of personalisation.

The scenarios were performed twice, according to the schedule shown in Table 2. They both required the participant to engage in a structured role play-like scenario [36] in order to investigate the role of the robot in a manner that could be directly related to the participant’s everyday experience, thus allowing the participant insight into the potential impact of the robot on their lives. In addition to high-level evaluation of the experience of using the robot in these scenarios, the scenarios also allowed the researchers to investigate particular issues that were of interest to the research theme, in particular the issues of communication and agent migration.

**Table 2:** Overview of experiments

Week	Session content
Week 1	Introduction to the Robot House, familiarisation with the robots and their interface. Baseline experiment
Week 2	Review of Robot House, robots and interface Repeat of experiment
Week 3	Open-ended Scenario A
Week 4	Open-ended Scenario B
Week 5	Repeat of experiment
Week 6	Open-ended Scenario A
Week 7	Open-ended Scenario B
Week 8	Repeat of experiment
Week 10	Debriefing

#### Scenario A: Morning and Delivery—Communication

This particular scenario was intended to investigate participants’ interactions with, and responses to, the robot in an everyday setting. In addition, this particular study was intended to investigate the role of attention-seeking and other expressive behaviours in the robot. The Sunflower robot that was used in the study is what can be described as “appearance-constrained”, or having an appearance that is constrained by required practical functions, rather than having been created for specific anthropomorphic communication modalities [37]. There are several situations that require expressive behaviours from a robot, and we have investigated several in experimental settings based on the UH Robot House, including attention [28, 31] and relationship–building cues [19]. In this scenario, we examined the perceived efficacy of these behaviours by integrating episodes which required the robot to attract the attention of the user. The participant’s briefing asked them to imagine that they had just woken up. The participant would then go to the sofa and be approached by the robot, which suggested one of three activities through messages on its touch screen: Making/drinking a hot drink and making/eating breakfast, or one of three leisure activities.

The specific activities and the type of drink and breakfast for each participant, were determined by their previously indicated preferences. Throughout these tasks, the robot would offer assistance by highlighting the appropriate location for the task, and then, using the sensors attached to the kitchen appliances it would inform the participants of when the kettle had boiled, toaster had popped or egg cooker had finished. In addition, while the participant was performing one of these tasks, the robot alerted the participant to the doorbell having been rung, as part of the episode in which the newspaper was being delivered. This episode was introduced in order to investigate the efficacy of the robot’s expressive behaviour. Once a participant had completed one of the three activities, there would be a delay of 5 min before the robot suggested the next activity. Once the third activity had been completed, the robot would wait for an additional 5 min and then display the option to end the session. If at any time the participant did not want to engage in any activity yet, the participants had the option to request that the robot waits for a set period of time before the next reminder.

#### Scenario B: Afternoon and Phone Call—Agent Migration

This scenario was intended to investigate participants’ impressions of the use of the robot in a situation that involved *agent migration*. Agent migration is a term describing the ability of an agent “mind” to move between different robot and virtual embodiments [38, 39]. This allows the agent to both take advantage of features and functionalities of more than one embodiment while maintaining the persistent features that make it unique and recognisable from a *user’s* perspective, such as awareness of interaction history and context, as well as persistent customisable features [40].

There are many benefits from such an ability, since it allows for a wider range of functions as the agent is not bound by the constraints of a single robot platform. However, implementing this functionality and using it in HRI experiments pose many technical challenges. There are also many salient issues from an interaction perspective, such as how the agent can retain its perceived identity across different embodiments and how the process of migration, both from an embodiment and into another, is signalled in the different embodiments.

In this scenario, the migration took part between a Sunflower and a SONY AIBO robot. Migration was indicated to the participants using the following signals:

- Sunflower
  - Migration into another embodiment:
    - Light comes on, “head” lifts up to the highest position and tilts once to each side before coming down to the default position.
  - Migration out of an embodiment:
  - Head moves back from default position and down, light switches off.
- AIBO
  - Migration into another embodiment:
    - AIBO lifts its head and stands up, lights come on.
  - Migration out of an embodiment:
  - AIBO lies down and puts head down, lights switch off.

The participants’ briefing asked them to imagine that it was the afternoon and they had just returned home. The participant sat on the sofa and was approached by the robot, suggesting two activities, watching TV or having a snack and a drink. The specific TV programme and snack and drink combination was based on their previously indicated preferences. As in Scenario One, the robot offered assistance by highlighting the appropriate location for the task and the specific TV programmes that the participant had previously indicated a preference for.

During this scenario, the activities of the participant were interrupted in order for them to use the AIBO for remote interactions. The Sunflower robot would approach the participant, to either inform them that they had a scheduled Skype call that they needed to make, or that there was an incoming Skype call that they needed to respond to. This Skype call involved a collaborative game that could be played over Skype and which used the AIBO embodiment. The game used was a social mediation game developed as part of a separate research strand and is described in [41]. This scenario was not intended to investigate the specifics of the social mediation game, rather the migration that it necessitated was the focus of this study, and the game itself was only evaluated as part of the entirety of the scenario.

After the Skype interaction was completed, the participant was free to return to their leisure activity. Unlike the “Morning” scenario, the incoming Skype call presented an event that the participant had to respond to, but all other activities could be delayed.

### Research Questions

The research questions for this study were concerned with the acceptability of the agent, both in terms of its usability as well as its role in the scenarios as a whole. We were also interested in how engaged the participants were with the agent and the scenarios throughout the study. Finally, we were interested in the transferability of these scenarios to the world outside of the research scenarios. Did the participants feel that the interactions they had with the agent in the UH Robot House were meaningful in terms of their everyday experience, and that the agent they interacted with, had a role to play in the lives of themselves and others?

Open-Ended Research Question 1 (Usability):

- Did the participants find the agent easy to use?

Open-ended Research Question 2 (Engagement):

- Did the participants find the agent and the scenario engaging over time?

Open-ended Research Question 3 (Acceptability):

- Did the participants accept the agent within the scenario?

Open-ended Research Question 4 (Transferability)

- Did the participants find the scenarios and interactions relevant to their own, everyday experience?

Open-ended Research Question 5 (Target Behaviours)

- How did participants evaluate the expressive behaviours of the agent?
- How did participants view migration and identity retention of the agent?

These questions were addressed by a series of measures detailed below.

## Method

### Apparatus

**Fig. 4:**
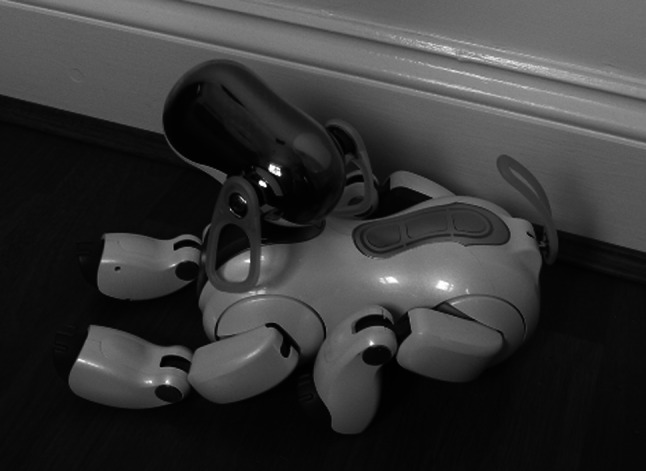
Sony AIBO robot

As mentioned previously, two different robots were used in this study. The first was the UH Sunflower robot, which uses a Pioneer base (commercially available from MobileRobots), but which has been modified significantly (see Fig. 1). The main mode of direct interaction with this robot is its touch screen which can be used to display information to the user and for issuing commands to the robot. Sunflower also has an extendable tray which can be used to carry light-weight objects. The second robot was a SONY AIBO as shown in Fig. 4. In addition, laptops were used to set up Skype calls.

### Interaction Setup

The sessions were performed as part of a long-term study taking place in the UH Robot House. As part of this study, participants were asked to visit the Robot House once a week for a period of 10 weeks in order to see how participants’ views of, and interactions with, the robots changed over time. Table 2 provides an overview of the sessions that the participants took part in. References made to a specific week of the experiment in this paper will be based on this table. Participants interacted within the open-ended scenarios in weeks 3, 4, 6 and 7. In the remaining weeks, however, participants still interacted with the robots, which allowed further familiarisation with the platforms.

**Fig. 5:**
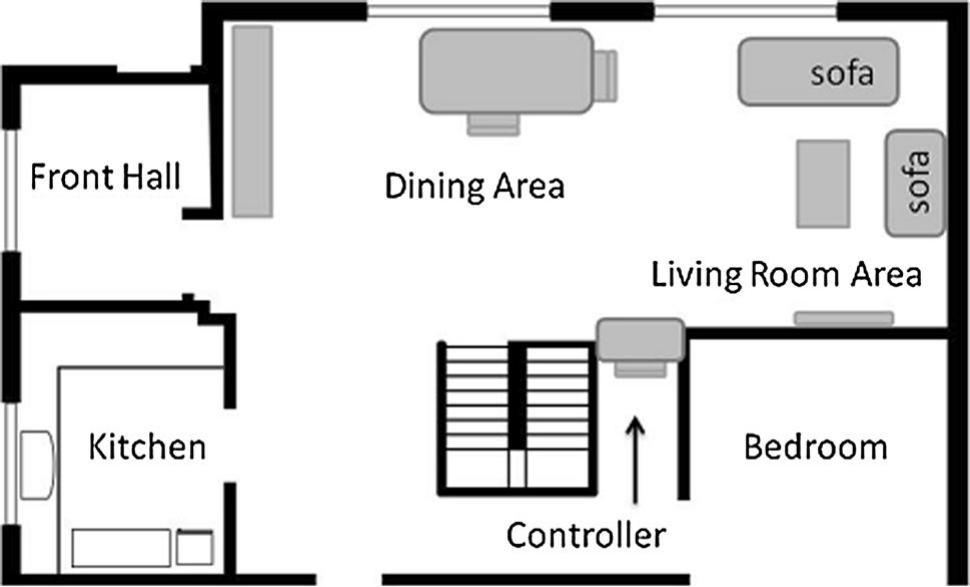
University of Hertfordshire Robot House Layout

The layout of the Robot House is shown in Fig. 5. The participant would normally begin each session on the sofa and would move to the kitchen, dining area and front hall throughout the scenario. For safety reasons, an experimenter was seated in the area beneath the staircase so that they could respond if necessary.

### Procedure

#### Introduction

The introduction session introduced the UH Robot House and the robots to the participants. In this session, the participants were instructed in the use of the Sunflower robot and its touch screen. They were also given an overview of how the robot responded to scheduled and sensor events. The participants were given a tour of the living areas that they would interact with the robot in and shown the kitchen cupboards and fridge shelves that would be “theirs”. Throughout this tour, participants were encouraged to think of these areas as their home and put themselves in the mindset of someone living in the house. This was intended to begin the process of framing the narrative [12] of the open-ended scenarios. Participants were given a chance to use the robot to perform tasks similar to that in Open-ended Scenario A and shown how to use the AIBO in the interactive game.

#### Constrained Experiments

In addition to the open-ended scenarios, participants were given the chance to interact with the Sunflower robot in a series of constrained experiments in weeks 1, 2, 5 and 8. These were clearly delineated from the open-ended scenarios and due to space considerations are not discussed in this article.

#### Open-Ended Scenarios

As mentioned in the introduction, there were two open-ended scenarios that were presented twice to the participants. At the beginning of each open-ended scenario session, the participants were given a narrative framing of the context of the scenario that they were taking part in, beyond that in the introductory session in which they were told the time of day, and what had immediately transpired before the beginning of the scenario.

*Scenario A: Morning* began in the morning and the participants were told the following

“Imagine that you have now woken up. In the introductory session, you gave us some preferences for what you would like to do in the early morning. The robot has these preferences and will try to help you do them. When you are ready, you will come out of the bedroom and sit down on the sofa. The robot will then approach you”.

*Scenario B: Afternoon* began in the afternoon

“Imagine that it is afternoon, and you have just returned home and have just sat down on the sofa. You have planned to watch some TV. In the introductory session, you gave us some preferences as to what TV programmes you like to watch and what sorts of snacks and drinks that you prefer to eat. The robot has recorded these preferences. It will also respond to events such as phone calls and doorbells. When you are ready to begin, sit down on the sofa and the robot will approach you.”

After this briefing, the scenarios ran as outlined in “From Usage to Evaluation Scenarios” section. Participants were asked to fill in questionnaires after the scenario was completed.

### Measures

The open-ended nature of the interactions makes it difficult to directly compare the experiences of the participants to each other. This made *pragmatism* and *inclusivity* in terms of measures used necessary. An overview of the measures used and the research questions they are intended to address can be found in Table 3.

**Table 3:** Research questions and measures

Research questions	System Usability Scale	Scenario Acceptance Scale	Ad hoc Likert Scales	Open-ended responses
Engagement				X
Usability	X			X
Acceptability		X	X	X
Transferability		X	X	X
Target behaviour			X	X

#### High-Level Measures

Evaluation of the robot’s behaviour was conducted via both quantitative questionnaires like the Simple Usability Scale [42] as well as an ad hoc questionnaire intended to measure the participants’ acceptance of the robot within the scenario (See “Appendix”). In addition, single-item questions were intended to assess the participants’ willingness to own and interact with a robot like this in their own everyday life, as well as the suitability of the robot for someone with particular needs and/or impairments.

These questions were also followed up by open-ended questions allowing participants to express their responses to the agent in an unconstrained manner. These questions were as follows:

- What was the best aspect of the agent in today’s session?
- What was the worst?
- Based on your experience in this interaction, would you like a robot like the ones you have interacted with in this session?
- Based on your experiences in this session, do you think this robot would be suitable for someone who is elderly or disabled?

In addition to the questionnaire measures, there was an unstructured interview at the end of week 10. These interviews were an attempt to address each individual’s subjective experience and get as wide a range of responses as possible. It was hoped that these responses would provide anecdotal data, offering explanations for observed quantitative effects as well as avenues for further investigation.

#### Target Behaviours

##### Expressive Behaviours

Participant responses to the Expressive Behaviours were measured using an ad hoc scale of 6 Likert questions intended to measure the following:

1. Saliency—How clear was it that the robot was signalling something?
  - It was easy to notice that the robot required my attention.
  - It was not clear that the robot wanted me to respond to something (negative)
2. Clarity—How clear was what the robot wanted to achieve?
  - It was clear where the robot wanted me to go
  - It was difficult to find out what the robot was drawing my attention to (negative)
3. Distraction—How distracting was the behaviour of the robot?
  - The attention-seeking behaviour did not stop me from going about my business.
  - The attention-seeking behaviour of the robot was distracting me from what I was doing (negative)

In addition, participants were invited to respond to the following open-ended questions:

1. How did you find the robot’s attention-seeking behaviour?
2. What was the best part about it?
3. What was the worst part about it?

##### Migration

Two aspects of the migration scenario were measured, *identity retention* and *migration signalling*. These were investigated using ad hoc Likert scale questions and open-ended questions.

1. Identity retention:
  - Did you feel as if you were interacting with the same agent in both embodiments?
2. Migration signalling
  - Was it clear when the mind of the body left the Sunflower embodiment?
  - Was it clear when the mind of the body entered the AIBO?

Participants were also invited to share their responses to the following two open-ended questions:

1. Did it feel as if you were interacting with the same companion across embodiments?
2. How should it better communicate that it is the same companion in both bodies?

### Participants

There were 12 participants in the study, recruited through advertisements on the University of Hertfordshire Intranet, mailing lists and social networks. There were 8 males and 4 females in the sample. The mean age was 32 and the median age was 26, and the range was 18–64.

## Results

The results for the open-ended scenarios are described first in terms of responses *to the measure used* beginning with the standardised measures, followed by the open-ended questionnaire items. Where appropriate, the results will reference the research questions of *Acceptability, Usability, Engagement and Transferability,* but the impact of these results on the specific research questions will be considered fully within “Summary of the Results” section.

### High-Level Measures

#### System Usability Scale

System Usability Scale results are presented in Table 4. There were no differences between the different sessions showing no effect of Scenario, also, there was no significant effect from long-term interaction either, although there was a small trend that participants rated the agent as easier to use in the later sessions. The scores were overall quite high, suggesting that participants did not find the *Usability* of the system to be an issue.

**Table 4:** System Usability Scale results by week

Scenario	Mean SUS (SE)	Median SUS	Difference from “neutral value”
Morning 1	71.9 (3.76)	67.5	21.9 (*t* = 5.82, *p* = .01)
Afternoon 1	70.8 (3.51)	71.3	20.8 (*t* = 5.93, *p* = .01)
Morning 2	73.0 (4.81)	75.0	23.0 (*t* = 4.78, *p* = .01)
Afternoon 2	73.6 (6.07)	72.5	23.6 (*t* = 3.89, *p* = .01)

#### Scenario Experience Scale, SES

The Scenario Experience Scale was an ad hoc scale and as such it was assessed using Cronbach’s alpha. It was found to have a high inter-item reliability (*α* = .91), suggesting that it reliably measured one underlying construct. The questions in this scale focused primarily on how the system worked within the framework in the session as well as drawing parallels to use in other environment and is presented in full in “Appendix”. The results are presented in Table 5. As for the SUS Scores, there were no effects from Scenario Type nor from Instance, but overall scores were high, suggesting that participants found both open-ended scenarios to be *Acceptable* and have a high degree of *Transferability* to their own everyday experience.

**Table 5:** Scenario Experience Scale by week

Scenario	Mean SES (SE)	Median SES	Difference from “neutral value”
Morning 1	3.81 (0.25)	3.89	0.81 (*t* = 3.18, *p* = .01)
Afternoon 1	3.84 (0.19)	4.00	0.84 (*t* = 4.43, *p* = .01
Morning 2	3.82 (0.26)	4.00	0.82 (*t* = 3.13, *p* = .01)
Afternoon 2	3.98 (0.22)	4.11	0.98 (*t* = 4.42, *p* = .01

#### Single-Item Questions

The responses to the single-item questions are presented in Table 6. There were no differences between Scenarios and Instances for the Robot for Self-item. For the Robot for Others-item, there was a significant main effect for Scenario (*F*(1,11) = 15.71, *p* < .005, *η* ^2^ = 0.61) and a main effect approaching significance for Instance *F*(1,11) = 4.81), *p* = .053, *η* ^2^ = 0.33). The effect for Scenario suggested that participants saw the “Morning” Scenario as more suitable for others, while the effect for instance suggested that participants saw the robots as more suitable for others in the two later instances, which suggested that the high degree of *Transferability* and *Acceptability* exhibited in the SES responses might not have been that clear cut, when considering the adoption of the robots into the participants’ own everyday life.

**Table 6:** Single-item questions by week

Scenario	Robot for self			Robot for others		
	Mean (SE)	Median	Distance from “neutral value”	Mean (SE)	Median	Distance from “neutral value”
Morning 1	2.58 (0.34)	3.00	−0.42 (*t* = 1.24, *p* = .24)	4.67 (0.14)	5.00	1.67 (*t* = 11.1, *p* = 01).
Afternoon 1	2.42 (0.29)	3.00	−0.58 (*t* = −2.03, *p* = .07)	3.92 (0.23)	4.00	0.92 (*t* = 4.01, *p* = .01)
Morning 2	2.46 (0.37)	3.00	−0.55 (*t* = 1.49, *p* = .18)	4.82 (0.12)	5.00	1.82 (*t* = 14.9, *p* = .01)
Afternoon 2	2.18 (0.26)	2.00	−0.82 (*t* = 3.11, *p* = .01)	4.18 (0.18)	4.00	1.18 (*t* = 6.50, *p* = .01)

#### Relationships Between Quantitative Measures

Correlations between the quantitative measures can be found in Table 7, which suggests that there are strong relationship between the SUS, SES and wanting an agent for oneself. This suggests that *Usability and Acceptability* are linked with both each other as well as perceived *Transferability.* The link between these measures and considering the agent suitable for others is less salient, and it does seem that participants contrasted the suitability of the agent for themselves with that of its suitability for elderly or disabled people. In addition, there seems to have been a ceiling effect in terms of how participants viewed the suitability of the agent for others, which may have limited the explanatory power of the other quantitative variables to explain the variance in scores along this variable.

**Table 7:** Spearman’s correlations between quantitative measures

	SUS	SES	Self	Other
*Morning 1*				
SUS	1.00			
SES	.68, *p* = .02*	1.00		
Self	.48, *p* = .12	.62, *p* = .04*	1.00	
Others	−.23, *p* = .4	−.12, *p* = .7	−.58, *p* = .05*	1.00
*Morning 2*				
SUS	1.00			
SES	.70, *p* = .02*	1.00		
Self	.41, *p* = .21	.72, *p* = .01*	1.00	
Others	.00, *p* = .99	−.15, *p* = .66	−.56, *p* = .07	1.00
*Afternoon 1*				
SUS	1.00			
SES	.74, *p* = .01*	1.00		
Self	.77, *p* = .01*	.81. *p* = .01*	1.00	
Others	.07. *p* = .83	.13, *p* = .69	.02, *p* = .96	1.00
*Afternoon 2*				
SUS	1.00			
SES	.97, *p* = .01*	1.00		
Self	.49, *p* = .13	.52, *p* = .10	1.00	
Others	−.35, *p* = .29	−.40, *p* = .22	−.02, *p* = .95	1.00

* *p* < .05

### Open-Ended Questions

Participant responses to open-ended questions are considered below regarding the responses to each question.

#### Open-Ended Question One: Best Aspect of the Agent

The responses to these questions across all four open-ended sessions were categorised into the following categories:

- Responses referencing the **reminder** functions of the agent:
  - “The robot reminded me that the water was boiled and the egg was cooked”
  - “The robot will remind you when toast and egg are cooked ok.”
- Responses referencing the idea of **companionship** from the agent:
  - “Act like a companion, will sit quietly while I was doing my own thing.”
  - “I liked having a companion. I felt like I was not on my own”
- Responses referencing the **novelty** of the interactions:
  - “Novelty of the situation”
  - “Something different to do”
- Responses referencing enjoyment or other **hedonic** aspects of the interaction:
  - “Quite good fun”
  - “The best aspect was when I was using the AIBO. The AIBO works really well and it is fun to use it”

The number of responses is shown in Table 8. This table suggests that participants considered the best aspects of the “Morning” Scenario to be that of the functional aspects of the robot while the hedonic/enjoyment aspect was considered the best part of the “Afternoon” Scenario.

**Table 8:** Responses to best aspect question

Scenario	Instance	Reminder	Companionship	Novelty	Hedonic	Missing
“Morning”	1	9	1	1	1	0
	2	8	2	0	1	1
“Afternoon”	1	1	1	1	7	2
	2	4	0	0	7	1

#### Open-Ended Question Two: Worst Aspect of the Scenario

The responses to all these open-ended questions were analysed across all four sessions, and the following categories were found:

- Responses referencing that there was **no negative** aspect to the interactions:
  - “No bad aspects”
  - “Nothing in particular, it was all good”
- Responses referencing the **slow speed** of the agent within the interaction:
  - “Was frustrating to have Sunflower direct me, waiting for her and the speed she moved at was annoying.”
  - “Waiting for Sunflower. too slow and too many “clicks” when doing anything”
- Responses referencing **difficulty**, either with the agent itself or with the scenario:
  - “The Agent made things more complicated”
  - “Sometimes the attention seeking was not clear to me. I was not able to clearly understand why it is telling me some things.”
- Responses referencing **technical errors**, either breakdowns or minor glitches:
  - “The robot got a little confused and kept asking me to go to places I already was in”.
  - “Sunflower freezing waiting for her when she was about to tell me stuff”

Participant responses according to these categories can be found in Table 9, which suggests that participants overall found the scenarios less negative in their second instance. The main cause for this seems to be that participants did not find the system as difficult to use in the second instances of the scenarios. Participants did, however, rate the agent as acting too slowly, and technical problems, when encountered, impacted their experience.

**Table 9:** Responses to worst aspect question

Scenario	Instance	No negatives	Slow speed	Difficult to use	Technical errors	Missing
“Morning”	1	3	3	3	3	0
	2	5	4	0	3	0
“Afternoon”	1	3	5	3	1	0
	2	6	1	0	5	0

When considered together, these responses suggest elements related to both *Usability* and *Engagement* were important to the participants in terms of what they found to be the best part of the interaction. The playful interactions that they had with the AIBO in the “Afternoon” Scenarios are here contrasted with the more practical interactions that they had in the “Morning” Scenarios. Concerning the worst part of the interactions, it seems that all the statements were directly related to *Usability*.

#### Open-Ended Question Three: Wanting the Agent

The open-ended responses to whether or not the participant wanted the agent were categorised into two main categories:

- Statements referencing **practical** concerns:
  - “Because it’s good to have robot like this to remind me of important things and for good time management.”
  - “The tasks are very easy to do. The robot is a bit superfluous at this level of activity”
- Statements referencing **emotional/hedonic** concerns:
  - “An amusing diversion”
  - “It’s fun to have a robot like this at home, it is a unique way of playing games.”

The frequencies of responses according to whether or not a participant wanted the agent can be found in Table 10. While both practical and emotional concerns figured in the reasoning for participants that wanted a robot or were neutral to the prospect, only practical concerns were considered for participants that did not want a robot.

**Table 10:** Open-ended responses to wanting a robot

Scenario	Instance	Wanting robot		Not wanting robot		Neutral	
		Emotional	Practical	Emotional	Practical	Emotional	Practical
“Morning”	1	1	5	0	3	1	2
	2	2	3	0	1	0	5
“Afternoon”	1	3	2	0	1	2	4
	2	3	5	0	0	2	1

#### Open-Ended Question Four: Finding the Robot Suitable for Others

Participant reasoning for whether or not the robot could be helpful was overwhelmingly dominated by the fact that participants all considered the robot to be potentially helpful to people with particular disabilities. All participants would reference specific disabilities that the robot would be of assistance with, including hearing problems and conditions that may cause problems with memory. A small group of participants did voice concerns that technical problems as well as price might be an impediment to adoption by users with specific disabilities, but despite this would rate the robot as highly suitable for someone who is elderly or disabled.

Both question three and four pertain directly to the issues related to *Acceptability* and *Transferability* of the interactions suggested in the responses to the single-item Likert questions. While statements related to both *Engagement* and *Usability* were predictive of positive responses to whether or not participants wanted robots, practical concerns (pertaining to the *Usability* of the agent) were the most important in negative statements. With regard to the suitability of the robot for the elderly or disabled, the statements were highly related to *Usability* both in terms of positive statements as well as negative.

### Target Behaviours

#### Expressive Behaviours

The ad hoc scale was examined for reliability using Cronbach’s alpha. After removing the two items intended to measure distraction, a Cronbach’s alpha of .75 was observed. This suggests that participants evaluated the expressive behaviour both in terms of clarity and salience. We considered this to be the perceived utility of the expressive behaviours, or the overall usefulness of the behaviour. The scores on this scale did not deviate significantly from the normal distribution (skewness = −.586, kurtosis = 1.7, K.S. = .559, *p* = .91). Scores on the expressive behaviours are presented in Table 11 and suggest that participants considered the robot’s expressive behaviour to be much clearer in the second instance of the scenario.

**Table 11:** Utility ratings of expressive behaviours

Week	Expressive behaviour utility rating		
	Mean (SE)	Median	Range
Morning 1	3.63 (.24)	3.75	1.75–5.00
Morning 2	4.34	4.25	3.50–5.00

#### Migration

The results for identity retention are presented in Table 12 and suggest that the participants consistently rated this as quite low throughout the study.

**Table 12:** Identity retention after migration

Week	Identity retention		
	Mean (SE)	Median	Range
Afternoon 1	2.25 (.30)	2.00	1.00–4.00
Afternoon 2	2.36 (.30)	2.00	1.00–4.00

When responding to the open-ended questions, only two participants in the entire sample expressed a feeling of having interacted with the same companion in both embodiments. Only one of these offered a reason, namely the clarity of the migration signalling between the embodiments. Participants who felt that the agent was not the same would reference both the differences in appearance, as well as interaction modalities as their main reason for not accepting the companion as “one mind in two bodies”.

There were some suggestions as to how the robot could better retain its identity in both embodiments. These included using similar signals, such as colours or sounds across both embodiments, or adding new modalities, like voice or written signals to suggest that the two embodiments housed the same agent. One participant, however, argued that it was impossible due to the differences between the AIBO and the Sunflower embodiments.

Migration signalling results are shown in Table 13. This suggests that participants rated the signalling of migration as less clear in the second instantiation of the episode, this trend was significant (*F*(1,10) = 5.45, *p* = .04) and most pronounced in the AIBO condition. Responses to open-ended questions suggested that this was due to their not paying as much attention to the robot’s behaviour, as they were focusing on the prospect of playing the game through the social mediator in the second session,

**Table 13:** Migration signalling

Question	Week	Migration signalling		
		Mean (SE)	Median	Range
Leaving Sunflower	Afternoon 1	3.8 (.27)	4.00	1.00–5.00
	Afternoon 2	3.36 (.36)	4.00	1.00–5.00
Entering AIBO	Afternoon 1	3.75 (.33)	4.00	2.00–5.00
	Afternoon 2	2.63 (.34)	3.00	1.00–4.00

In the post-trial interviews, participants most commonly referenced confusion during the call situations, and when playing the social mediator game. The participants suggested that the cognitive load of answering/making calls and playing the game led to less attention to the migration process. Another point made by the participants was that the disconnect between the interactions with the Sunflower robot and those with the AIBO was so large that it was difficult to reconcile these two.

### Anecdotal Observations

The following are anecdotal observations made by the researchers during the experiments, debriefs and viewing of the videos. They are anecdotal and only intended to illustrate the insights that this approach can give researchers and participants when using this kind of prototyping.

#### “Would you Like a Cup of Tea?”: The Role of Ownership

While we attempted to make the “ownership” of the Robot House more clear cut compared to earlier studies (see the “Previous Work in the Robot House” section), the role of “ownership” of the house was one of the more difficult things to understand, not only from the perspective of the participants, but initially also from that of the researchers.

One of the ways in which this manifested itself was in the role of offering tea. In most British households, a guest is customarily offered tea upon entry. This was also the case for the participants in the introductory session as well as the first constrained experiments, when the researchers would offer the participants tea when entering the UH Robot House, or when they were filling out (the often all-too-lengthy) questionnaires. However, it was decided not to offer this at the beginning of the open-ended scenarios. At the time, this decision was made for purely practical reasons; after all, the participants would likely get themselves a drink as part of their interaction with the open-ended scenario, but one of the researchers reported feeling uncomfortable about not being “hospitable” when not offering a drink to the participants through the course of the initial open-ended scenario. After some discussion, it was decided to use this behaviour actively, in order to mark the end of, or departure from, the open-ended scenarios. Participants would always be offered tea or coffee when entering the UH Robot House for constrained experiments and when filling out questionnaires after the completion of an open-ended scenario. This allowed another means of demonstrating the change of “ownership” and served to delineate the narrative space of the scenarios from the context of a HRI study. While it is difficult to gauge the effectiveness of such an individual measure, however, in the debrief session (S10), several of the participants commented on who was making the tea in that particular session, with one participant even offering tea to the experimenter before the experimenter had a chance to offer it.

#### “Come Here, Boy!”: Playing Pet-Like Interactions

Despite the fact that we attempted to frame the robot in terms of it being a piece of technology, many participants would sometimes behave towards it in a social manner (similar to phenomena reported in Reeves and Nass [43]) What was interesting is that this often took part outside of the touch-screen interactions where information was exchanged between the user and the robot. Often participants would turn to the robot when moving from one section of the robot house to another and encourage it to come towards them, sometimes slapping their legs and saying things like “Come here, boy!” or “Good robot!”. When asked about this in the debrief interviews, some participants responded that they felt the robot was a bit slow, and they would be bored waiting for the robot, so amused themselves by talking to the robot. This phenomenon is in line with the findings of Luczak et al. [44] who found that people often use such anthropomorphising behaviour to cope with dissatisfaction with technological devices. What is interesting, however, is that the verbal utterances of the participants seemed to cement a relationship between the participant and the robot as pet-like. The playful nature of this behaviour is also similar to the performed belief–behaviour suggested by Jacobsson et al. [6], which in turn suggests that the technique of narrative framing gave these participants licence for further play–exploration of the HRI scenarios in this study.

## Summary and Discussion

### Summary of the Results

#### Open-Ended Research Question One: Engagement

As suggested in the “Open-Ended Questions” section, statements related to *Engagement* were primarily found when participants were discussing the “Afternoon” Scenario. It seems that this dimension was not considered as important for the “Morning” Scenario. In addition, participants would reference only engagement-related reasoning when considering positive aspects of the interaction, not as a negative. For instance, the absence of *Engagement* with the agent was never referred to by the participants when discussing negative aspects.

#### Open-Ended Research Question Two: Usability

Participants did overall rate the usability of the system as quite positive. The findings presented in the “System Usability Scale” section show responses to the SUS which were significantly higher than the “Neutral” value of 50, suggesting that overall the participants found the system usable and helpful in terms of carrying out their tasks within the context of the scenarios.

Statements related to the usability of the system reported in the “Open-Ended Questions” section were used in the reasoning of the participants when considering both positive and negative aspects of the scenarios. They were most pronounced when discussing the suitability of the system for elderly or disabled people. Even more salient was the fact that *Usability* was the only aspect referenced for negative aspects of the interactions, as well as in reasoning explaining why the agent was not suitable for the participants themselves or others.

#### Open-Ended Research Question Three: Acceptability

Overall, participant responses suggest that the group viewed the agent and the interactions with it as quite acceptable. The results reported in the “Scenario Experience Scale, SES” section show that participants overall scored significantly higher than the “Neutral” value of three on the SES scale. This suggests that the scenarios were acceptable in and of themselves. There was a clear quantitative and qualitative link between the participants’ acceptance of the scenarios and the experienced *Usability* of the system. In addition, the findings in the “Open-Ended Questions” section suggest that there was a qualitative link between *Engagement* and *Acceptability* for the “Afternoon” scenario.

#### Open-Ended Research Question Four: Transferability

*Transferability* was a less clear-cut issue for these scenarios. While participants rated the system as highly *Usable* and found its use in the scenario *Acceptable*, there was a trend, reported in the “Single-Item Questions” section in which the participant responses to the Likert item regarding whether or not they wanted such a system in their own lives were less than the “Neutral” score of 3. This trend was significant for the “Afternoon” Scenarios. In terms of *Transferability* to the participant’s own lives, the correlations reported in the “Relationships Between Quantitative Measures” section suggest that both *Usability* and *Acceptability* seemed to be important in terms of quantitative responses. However, the open-ended responses in the “Open-Ended Question Three: Wanting the Agent” section suggest that *Engagement* was also important for the “Afternoon” Scenario, when it came to justifying a high degree of *Transferability* to the participant’s own lives. When participants were justifying a low degree of *Transferability*, however, they would highlight usability issues, either difficulties they had with using the robot for specific tasks or a lack of need for the agent’s help.

Perceived *Transferability* to elderly or disabled people’s lives as suggested by the correlations in the “Relationships Between Quantitative Measures” section seemed either orthogonal or negatively correlated with the participants’ own lives. For the “Morning” scenarios, in particular, it seems as if the participant directly contrasted the use of the robot for their own lives with that of its use for others.

#### Open-Ended Research Question Five: Target Behaviours

In terms of specific behaviours, the results in the “Expressive Behaviours” section suggest that participants found it easier to relate to and understand the expressive behaviours of the robot over time, but the opposite was true for the responses reported in the “Migration ” section concerning the agent migration behaviours. The open-ended responses to questions regarding the migration behaviour suggested that this might have been an artefact of the difficulty of the game itself, rather than what we intended to measure.

This suggests that the use of the AIBO game as a means to investigate migration between robot embodiments was not completely successful. While Papadopoulous et al. [41] suggest that this game is easily learned by a wide range of participants in a study solely evaluating the game, it seems that for our scenarios in the Robot House, where the game was integrated in an already elaborate scenario, the added cognitive load of having to learn a different set of interaction modalities became the focus of the migration episodes rather than the migration itself.

## Discussion

The findings suggest that overall, participants were able to consider various aspects of the interaction scenarios in a way that was meaningful to them. In particular, the relationship between *Usability*, *Engagement* and *Acceptability* found in this article complements the results from studies of users of simple consumer robots, such as the Roomba (Sung et al. [4]), which found that owners of these robots find the use of these robots pleasurable beyond their labour-saving capabilities. This suggests that while the ability to use such a system effectively and allow it to make everyday tasks easier is key for its adoption, the interactional aspects of the system, the fun of using it, the companionship, and, of course, the novelty that it may provide, is also something that will make it more acceptable to its user. The findings from this study suggest that this is likely also the case for more complex systems such as the one presented in these scenarios.

There was, however, one aspect of the study which while limiting the applicability of the study to the intended usage outcomes of the LIREC and ACCOMPANY projects, may explain some of the relationships between the different scenarios and the participants’ evaluations. The sample used was taken from a general adult population, which might have explained the differences in how participants reasoned about the transferability between their own lives and that of a disabled/elderly other. This might have made it more easy to define these users by their needs based on disabilities or illnesses rather than other personal characteristics, which led participants to justify the adoption of the system for this purpose rather than hedonic purposes. This is despite the fact that a game-like interaction played a large part in the “Afternoon” Scenario, the hedonic value of which some participants saw as quite important to their own acceptance and the perceived transferability of the scenario. A related explanation is that, as none of the participants did actually need any assistance to go about their daily lives, those of the participants who did see a place for the agent in their own lives, would justify it in terms of the hedonic value that the agent could add to their lives. While the use of an adult population was justified in terms of the basic nature of the research addressed in the paper, a more application focused investigation should endeavour to use a sample representative of the intended user-group.

However, our results show that our approach, i.e. a prototyping method using the narrative framing technique, is promising and may be applicable to a wide range of other human-centred settings and environments. Our work focuses on using narratives to set-up interactions in domestic environments, but the prototyping approach of using task- and persona-based scenarios to create realistic narratives may also be applicable to other environments, e.g. robots operating in hospitals and museums. The main challenge in applying this methodology to different settings is the integration of technical constraints with meaningful scenarios.

## Conclusions

The work presented in this paper suggests that our approach to high-fidelity episodic prototyping where narrative framing is used to provide context, while using the UH Robot house to support this narrative, is promising. Participants felt able to assess and respond to robot behaviours in a manner that they felt was grounded in a reasonable understanding in how such a system would impact their everyday lives. The findings from this study also are in support of Dautenhahn’s thesis that human–robot interaction need to be pragmatic in terms of data capture [11]. Both qualitative and quantitative measures as well as observations of emergent behaviours were valid sources of insight and allowed us to examine the interactions from different angles. However, the study did highlight the issue of cognitive overload, in terms of how participants experienced the change of embodiment. While the use of narrative framing was successful at conveying the *impression* of a long-term interaction when the participants were interacting with the Sunflower robot, it did not confer the *mastery* that was needed to address the experiences surrounding the complex interactive game played with the AIBO, and so our intended focus on agent migration to different embodiments could not be addressed in a satisfactory manner. Future investigations using this type of methodology need to consider this dichotomy between experience and mastery and use other techniques for more cognitively demanding tasks.

However, the use of the narrative framing techniques outlined in this article allowed us to examine a rich set of human–robot interactions. The high-fidelity robot prototypes in a realistic environment were effective in aiding the narrative framing of the interactions, allowing the participants not only to play along, but to put themselves into the narrative, and interact with an emergent technology in a robust manner.

While this article reported on results from a specific human–robot interaction study, the narrative framing techniques are potentially applicable over a wide range of different human–robot interaction studies that require high-fidelity interaction prototyping.

## Appendix: Scenario Evaluation Scale

Here, are some more questions about the use of the companion in this scenario. Please answer them based on your experience in today’s session as a whole. Please consider both of the embodiments you interacted with when answering the questions.

**Table:**

Statement	Strongly disagree	Disagree	Neutral	Agree	Strongly agree
I enjoyed using the robots in this session					
I don’t think I having a robot like this would be useful for me in my home					
I felt that the robots helped me do what I was meant to do in this session					
I found using the robots quite stressful in this session					
I think I would like using robots like these for tasks such as this in my own home					
I felt that the robots made the tasks that I was meant to do in this session more difficult					
I think that robots like this would be helpful for me in my own home for tasks like this					
I don’t think that having robots like this in my home would be particularly enjoyable					
I felt very confident using the robots in this session					
I needed to learn a lot of things before I could get going with the robots as I did in this session					
